# Maintenance therapy with toceranib following doxorubicin-based chemotherapy for canine splenic hemangiosarcoma

**DOI:** 10.1186/s12917-015-0446-1

**Published:** 2015-06-11

**Authors:** Heather L. Gardner, Cheryl A. London, Roberta A. Portela, Sandra Nguyen, Mona P. Rosenberg, Mary K. Klein, Craig Clifford, Douglas H. Thamm, David M. Vail, Phil Bergman, Martin Crawford-Jakubiak, Carolyn Henry, Jennifer Locke, Laura D. Garrett

**Affiliations:** Departments of Veterinary Biosciences and Veterinary Clinical Sciences, College of Veterinary Medicine, The Ohio State University, 454 VMAB, 1925 Coffey Rd, 43210 Columbus, OH USA; Department of Veterinary Clinical Medicine, University of Illinois at Urbana-Champaign, College of Veterinary Medicine, Champaign, IL USA; Animal Referral Hospital, Sydney, Australia; Veterinary Cancer Group, Tustin, CA USA; Southwest Veterinary Oncology, Tucson, AZ USA; Hope Veterinary Specialists, Malvern, PA USA; Department of Clinical Sciences, College of Veterinary Medicine and Biomedical Sciences, Colorado State University, Fort Collins, CO USA; Department of Medical Sciences, School of Veterinary Medicine, University of Wisconsin, Madison, WI USA; VCA Katonah Bedford Veterinary Center, Bedford Hill, NY USA; Sage Centers for Veterinary Specialty and Emergency Care, Concord, CA USA; Department of Veterinary Medicine and Surgery, College of Veterinary Medicine, University of Missouri, Columbia, MO USA; Southeast Veterinary Oncology and Medicine, Orange Park, FL USA

**Keywords:** Hemangiosarcoma, Toceranib, Dog, Chemotherapy

## Abstract

**Background:**

Spenic hemangiosarcoma (HSA) in dogs treated with surgery alone is associated with short survival times, and the addition of doxorubicin (DOX) chemotherapy only modestly improves outcome. The purpose of this study was to evaluate the impact of toceranib administration on progression free survival in dogs with stage I or II HSA following splenectomy and single agent DOX chemotherapy. We hypothesized that dogs with splenic HSA treated with adjuvant DOX followed by toceranib would have prolonged disease-free interval (DFI) and overall survival time (OS) when compared to historical dogs treated with DOX-based chemotherapy alone.

**Results:**

Dogs with stage I or II splenic HSA were administered 5 cycles of single-agent DOX every 2 weeks beginning within 14 days of splenectomy. Dogs were restaged 2 weeks after completing DOX, and those without evidence of metastatic disease began toceranib therapy at 3.25 mg/kg every other day. Forty-three dogs were enrolled in this clinical trial. Seven dogs had evidence of metastatic disease either before or at re-staging, and an additional 3 dogs were found to have metastatic disease within 1 week of toceranib administration. Therefore 31 dogs went on to receive toceranib following completion of doxorubicin treatment. Twenty-five dogs that received toceranib developed metastatic disease. The median disease free interval for all dogs enrolled in this study (*n* = 43) was 138 days, and the median disease free interval for those dogs that went on to receive toceranib (*n* = 31) was 161 days. The median survival time for all dogs enrolled in this study was 169 days, and the median survival time for those dogs that went on to receive toceranib was 172 days.

**Conclusions:**

The use of toceranib following DOX chemotherapy does not improve either disease free interval or overall survival in dogs with stage I or II HSA.

## Background

Hemangiosarcoma (HSA) is one of the more common malignancies that arise in dogs, accounting for approximately 20 % of soft-tissue sarcomas and 5 % of all non-cutaneous tumors [1, 2]. HSA can occur in several different organs including the spleen, liver, heart, muscle, retroperitoneal space and subcutaneous tissues. In some cases, multiple primary tumors may be evident at presentation. In nearly all cases, metastatic disease is present at the time of diagnosis, either in the form of microscopic or macroscopic lesions. Consequently, the majority of dogs diagnosed with hemangiosarcoma die within 1 year of diagnosis, with a large proportion of dogs not surviving beyond 6–8 months [2]. With respect to HSA involving the spleen, surgery alone has been associated with extremely short survival times ranging from 1 to 3 months [3–5]. The addition of doxorubicin (DOX)-based chemotherapy protocols (DOX alone, DOX/cyclophosphamide and vincristine/DOX/cyclophosphamide) following surgery has resulted in modest increases in median survival time (5–6 months on average), however the 1 year survival rate is 10 % at best [6–11].

Several clinical trials have been pursued in an effort to improve outcome in dogs with HSA, and most of these have focused on the splenic form as resection down to microscopic disease can be readily accomplished in many cases. The first of these involved the use of liposome encapsulated muramyl tripeptide phosphatidylethanolamine (L-MTP-PE), an immunomodulator that increases monocyte tumoricidal activity [12]. This was administered to dogs with DOX/cyclophosphamide chemotherapy following surgery, and a significant increase in disease free survival and overall survival time were noted compared to dogs receiving DOX/cyclophosphamide with placebo liposomes, although this was restricted to dogs that presented with Stage I disease (non-ruptured spleen). Unfortunately, dogs with Stage II disease (ruptured spleen) did not appear to derive significant benefit.

Another clinical trial involved the administration of a novel HSA vaccine prepared with lysates of allogeneic canine HSA cell lines mixed with an adjuvant composed of liposome-DNA complexes to dogs with splenic HSA following surgery [13]. Although vaccinated dogs mounted strong humoral immune responses against a control antigen and most dogs also mounted antibody responses against canine HSA cells, this did not translate into an improvement in survival time for treated dogs. The use of a metronomic treatment protocol consisting of piroxicam, low dose cyclophosphamide and low dose etoposide was evaluated in dogs with splenic HSA [14]. The therapeutic regimen did seem to have equivalent efficacy compared to DOX based studies, with no significant overall survival advantage noted as the median survival time was still approximately 6 months. More recently, intracavitary pegylated liposome encapsulated DOX was used to treat dogs following splenectomy and compared contemporaneously to single agent DOX; again, no advantage of the liposomal formulation was noted [15].

Given the failure of standard chemotherapeutic approaches and immunotherapy to significantly alter outcomes, the potential for the use of more targeted therapies for HSA has been investigated using both in vitro approaches and mouse models of disease. Canine HSA cell lines and tumor samples have been shown to express the receptor tyrosine kinases stem cell factor receptor (KIT), platelet derived growth factor receptor (PDGFR) and vascular endothelial growth factor receptor (VEGFR) family members [16–23]. Other cell signaling elements found to be expressed and activated include focal adhesion kinase (FAK), SRC, and several members of the mTOR pathway [24–26]. The small molecule masitinib which blocks function of KIT and PDGFR inhibited the proliferation and induce apoptosis in canine HSA cell lines in vitro, although drug concentrations necessary for this effect ranged from 8.5–10.6 uM [19]. This is higher than the Cmax achievable in healthy beagle dogs (1.3–1.5 uM) [27]. More recently, both imatinib and the multitargeted small molecule inhibitor dasatinib (blocks KIT, PDGFR and SRC) demonstrated activity against HSA cell lines in vitro, and imatinib significantly reduced growth of canine HSA xenografts in mice [16]. Together, these data support the notion that inhibitors of the KIT, PDGFR, and/or VEGFR family members may exhibit biologic activity in dogs with HSA.

Toceranib phosphate (Palladia®, Zoetis Inc) is a multitargeted small molecule inhibitor that blocks signaling of KIT, PDGFR and VEGFR family members [28]. Toceranib has demonstrated activity against multiple tumor types including mast cell tumor, nasal carcinoma, apocrine gland anal sac adenocarcinoma and osteosarcoma, among others [28–30]. The purpose of this prospective clinical trial was to evaluate the impact of toceranib administration on progression free survival in dogs with stage I or II HSA following splenectomy and single agent DOX treatment.

## Methods

### Eligibility

This clinical trial was approved by the Clinical Research and Advising Committee at the College of Veterinary Medicine and Institutional Animal Care and Use Committee (IAUCUC) at Ohio State University; IACUC or similar approval was also obtained at all other participating academic and private practice veterinary centers. Dogs with histologically confirmed stage I or II splenic HSA that had undergone splenectomy were considered eligible for this clinical trial. Surgical biopsy of the liver, lymph nodes and mesentery were not required unless there was clinical suspicion of metastatic disease at the time of surgery. Prior to enrollment dogs underwent a series of tests including complete blood count (CBC), biochemistry profile, urinalysis, abdominal ultrasound, and thoracic radiographs. Dogs with no obvious evidence of metastatic disease on diagnostic imaging were eligible for enrollment. Additional eligibility criteria included age of at least 1 year, ECOG performance score of 0–1, adequate organ function as indicated by routine bloodwork (e.g., liver transaminases ≤ 3x the upper limit of normal (ULN), creatinine ≤ 1.5x ULN), and no evidence of any serious systemic disorder (e.g., cardiac disease) considered incompatible with the study. Dogs were required to enroll and begin chemotherapy within 14 days of splenectomy.

### Drug product and concomitant medication

Toceranib phosphate was provided by Zoetis (Madison, NJ) in 10, 15, and 50 mg size tablets. Concomitant medications considered acceptable for use to prevent and/or treat drug related toxicities included famotidine, omeprazole, metronidazole, loperamide, metoclopramide, ondansetron, maropitant, tramadol, carprofen, meloxicam and prednisone.

### Study design

A total of 43 dogs were enrolled in this study. All dogs were treated with 5 cycles of single-agent DOX (30 mg/m^2^ IV) every 2 weeks beginning within 14 days of splenectomy. All dogs were re-staged with abdominal ultrasound and 3-view thoracic radiographs 2 weeks after completing the final DOX treatment, and those dogs considered free of metastatic disease received toceranib at 3.25 mg/kg every other day (EOD). Dogs were evaluated at weeks 1, 2 and 4 following initiation of toceranib therapy and then every 4 weeks thereafter. A CBC was performed at every visit; serum biochemistry profile and urinalysis were performed every 8 weeks. Re-staging consisted of 3-view thoracic radiographs and abdominal ultrasound every 8 weeks. Dogs were evaluated for adverse events (AEs) at every study visit. AEs were defined and graded according to the published VCOG-CTCAE criteria [31].

### Assessment of progression free survival

Dogs were evaluated every 8 weeks following initiation of toceranib therapy for evidence of metastatic disease using both clinical evidence of disease (anemia, obvious hemorrhage, seizures) and diagnostic imaging (thoracic radiographs and abdominal ultrasound). If lesions were identified via imaging that were consistent with possible metastatic disease but could not be confirmed through cytologic analysis, these were followed closely. If the lesions were subsequently deemed to be true metastatic disease based on progression as assessed by repeat imaging, cytologic confirmation, or clinical signs (e.g., hemoabdomen, seizures consistent with brain metastasis), the date that the lesions were first noted was considered the true date of progression, consistent with current RECIST recommendations. This also applied to dogs in which clinical signs were present that were consistent with metastatic disease (e.g., seizures) which were later confirmed with either additional imaging or progression of signs. Recheck exams continued for a total of 12 months from the time of surgical removal of the spleen, after which time the study ended and follow-up recommendations were provided at the discretion of the attending clinician.

### Statistical analysis

Thirty-eight dogs were to enter this multi-center, single-arm trial over 18 months, with an additional 6 months for follow-up. It was anticipated that 6–8 of these dogs would fail chemotherapy and not be eligible to continue on to toceranib treatment, with a minimum of 30 dogs continuing on to receive toceranib therapy following restaging. A historical control group was used for the power analysis in the current study. This control group consisted of 21 dogs treated with stage I or II splenic HSA treated with splenectomy and single agent DOX every 2 weeks (unpublished data). One of the co-authors of this study (D. Thamm) provided the raw data from the dogs for the power analysis. Assuming a significance of 0.05 this study was powered to detect a 2-fold increase in median disease free interval or hazard ratio with 80 % power if 30 dogs went on to receive toceranib treatment following completion of DOX chemotherapy. A total of 38 dogs were planned for enrollment assuming attrition due to metastatic disease identified either during or immediately following completion of DOX treatment. An additional 5 dogs were added to the initial 38 anticipated for a total of 43 dogs entered; this was secondary to a higher than anticipated number of dogs having metastatic disease identified either prior to toceranib therapy or immediately thereafter. Disease-free interval (DFI) and survival time (ST) were calculated using the Kaplan-Meier method for all dogs on an intent to treat basis and for only those dogs that went on to receive toceranib following completion of DOX therapy.

## Results

### Demographics

This was a multi-institutional study, with dogs enrolled from multiple sites. A total of 43 dogs were enrolled from April 2010 through June 2011. Baseline demographic information for this patient population is presented in Table 1. The median age of all dogs entered was 10 years and the median weight was 29 kg. Golden retrievers (*n* = 13) were the most commonly represented breed.

**Table 1:** Patient demographics

**Age (years)**	
Mean	9.9
Median	10
Range	5–12
**Weight (kg)**	
Mean	26.7
Median	29
Range	7–48.2
**Breed**	
Pure Breed	29
Mixed Breed	14
**Gender**	
Male intact	2
Male neutered	29
Female intact	0
Female spayed	12
**Clinical Stage**	
I	5
II	38
**Completed Study**	
Yes	7
No	36

### Outcome

Seven dogs (16 %) (*n* = 2 stage I; *n* = 5 stage II) failed doxorubicin either before or at re-staging having obvious metastatic disease in either the abdomen (*n* = 5) or chest (*n* = 2). An additional 3 dogs (*n* = 2 stage I; *n* = 1 stage II) were found to have metastatic disease shortly after toceranib administration (brain *n* = 2, abdomen *n* = 1). In these cases, metastasis was already suspected due to lesions identified on ultrasound. However, metastasis could not be definitively confirmed until a bleeding event or the presence of clinical signs at toceranib initiation were subsequently found to be secondary to tumor spread (e.g., seizures, *n* = 2). Thus, 4 of 5 dogs with stage I HSA were removed from the study either prior to or soon after starting toceranib due to metastatic disease. One dog was removed within 1 week of toceranib administration due to owner non-compliance and another dog was removed from the study within 1 week of toceranib therapy due to a severe protein losing nephropathy. Therefore 31 dogs went on to receive toceranib following completion of doxorubicin treatment. The window of time between surgery and restaging prior to starting toceranib ranged from 74–99 days (median 84 days).

**Fig. 1:**
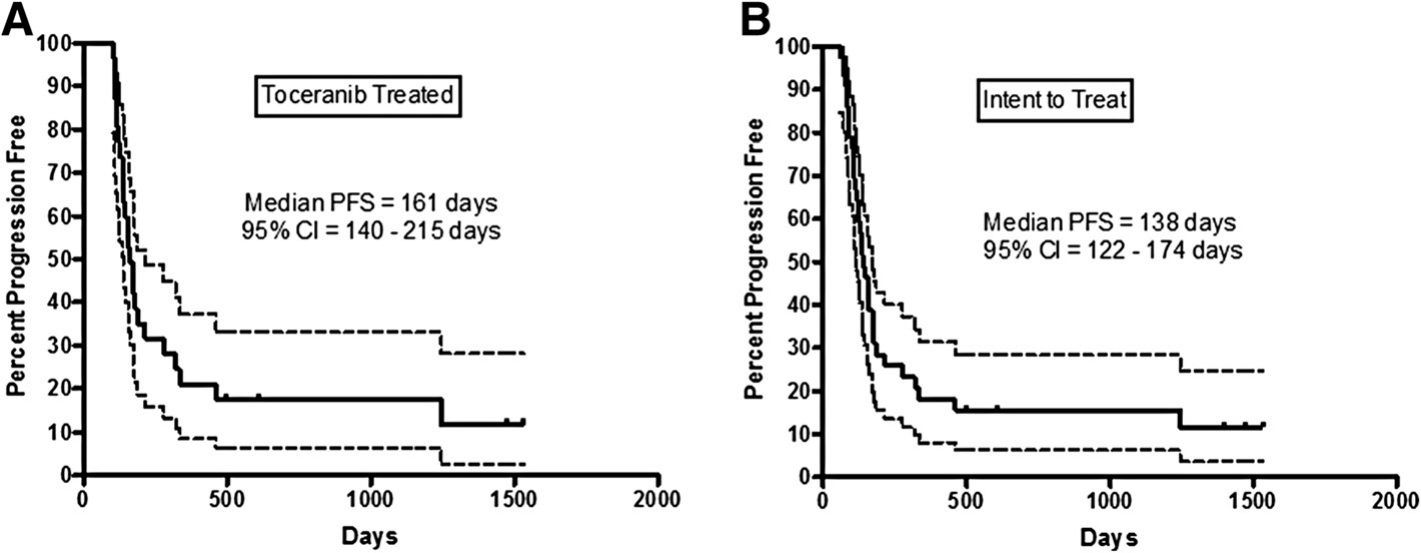
Disease Free Interval: Kaplan-Meier disease-free interval (DFI) curves for dogs entered into the clinical trial. Hash marks denote censored observations. *Dotted lines* delineate the 95 % confidence interval. (**a**) Dogs that received toceranib for longer than 1 week. (**b**) All dogs

**Fig. 2:**
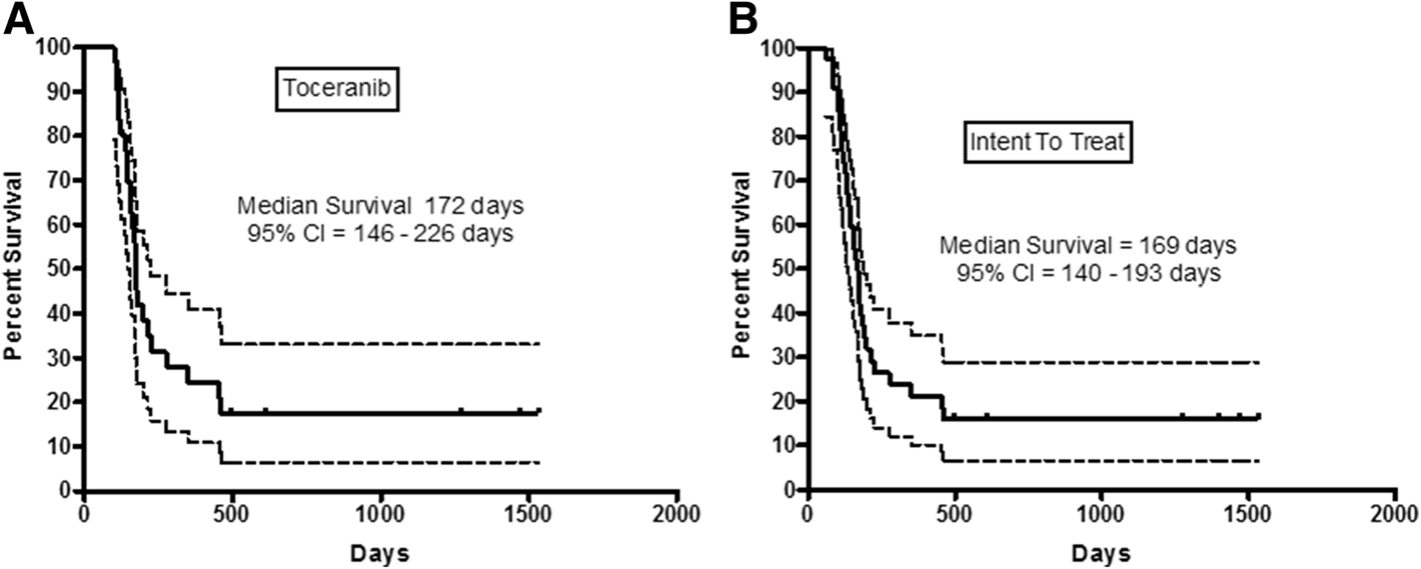
Overall Survival: Kaplain-Meier overall survival (OS) curves for dogs entered into the clinical trial. Hash marks denote censored observations. *Dotted lines* delineate the 95 % confidence interval. (**a**) Dogs that received toceranib for longer than 1 week. (**b**) All dogs

The majority (*n* = 25) of dogs that received toceranib developed metastatic disease. In most cases, this involved the development of metastasis in the abdomen (*n* = 16, liver, omentum, mesentery), lungs (*n* = 1), or abdomen and lungs (*n* = 3). Other sites of metastasis included the heart, bladder, brain, skin and kidney. Seven dogs completed the study and were alive 1 year post splenectomy (*n* = 1 stage I; *n* = 6 stage II). The median disease free interval for all dogs enrolled in this study (*n* = 43) was 138 days, and the median disease free interval for those dogs that went on to receive toceranib (*n* = 31) was 161 days (Fig. 1). The median survival time for all dogs enrolled in this study was 169 days, and the median survival time for those dogs that went on to receive toceranib was 172 days (Fig. 2). The 1 year survival rate was 21.2 % for all dogs enrolled, and 24.4 % for dogs that received toceranib. These data are consistent with prior publications evaluating both chemotherapy and/or investigational therapies in dogs with stage I or II HSA [6–8, 10–12].

### Toceranib dosing

Dogs were initially assigned a dose of 3.25 mg/kg; however the actual dose of toceranib phosphate administered was adjusted as needed for alterations in weight and AEs. Fifteen dogs required a dose adjustment. The median dose of toceranib administered over this study was 3 mg/kg (range 1.7–3.25 mg/kg).

### Adverse events

Adverse events are described for the 31 dogs that went on to receive toceranib. AEs were similar to those previously reported for doxorubicin and toceranib. All drug-related AEs were self-limiting and responsive to supportive care. Gastrointestinal AEs consisting of vomiting, diarrhea and anorexia were the primary drug-related toxicities observed. Table 2 shows adverse events for all dogs that received toceranib.

**Table 2:** Adverse events in toceranib treated dogs

Adverse event	Grade				
	1	2	3	4	5
Vomiting	38	2	2		
Diarrhea	32	9	2		
Decreased HCT (Anemia)	26	13	2	2	
Inappetance	20	6	3		
Decreased hemoglobin	17	5	2	1	
Weight Loss	11	1			
Neutropenia	8	3			
Increased CK	8				
Lethargy	6	7		2	
Elevated BUN	6	2	1	1	
Alopecia	5	1			
Hind limb weakness	4	3	2		
Hypoalbuminemia	3				
Nausea	3				
Lameness	1				
Trembling hind limbs	1				
GI blood loss	1	2			
Stiffness		3			
Pancreatitis		2			
Generalized weakness		1		3	
Hemoabdomen		1		6	3

#### Hematologic

Low-grade anemia was prevalent throughout the study, with 25 of 31 dogs (80 %) developing at least one episode of grade 1 or 2 anemia. In all cases, grade 3 or 4 anemia was secondary to progressive disease and occurred in association with hemoabdomen. No dose adjustments or temporary drug discontinuation were necessary for Grade 1 or 2 transient neutropenia.

#### Biochemical

Low-grade elevations in BUN and hypoalbuminemia were the most common biochemical toxicities. One dog each developed a grade 3 and 4 elevation in BUN without a concurrent increase in creatinine. No evidence of hepatotoxicity was noted in dogs enrolled in this study.

#### Gastrointestinal

Grade 1 and 2 inappetance, vomiting and diarrhea resolved with supportive care, temporary drug discontinuation, and/or drug dose modifications. Grade 3 inappetance was associated with progressive disease in all dogs (*n* = 3). Grade 1 and 2 weight loss was consistent with reported inappetance in dogs.

#### Neuromuscular

Grade 3 hind limb weakness was associated with progressive disease in all dogs. Grade 4 generalized weakness, which was associated with progressive disease in 2 dogs and attributed to metronidazole toxicity in 1 dog.

#### Other Adverse Events

Other side effects observed in dogs enrolled in this study included: increased creatinine kinase, lameness, hind limb trembling, stiffness, alopecia, pancreatitis and hemoabdomen. These adverse events could not be directly attributed to toceranib therapy, and may have been secondary to progression of disease or other unrelated causes.

## Discussion

Hemangiosarcoma (HSA) is a relatively common malignancy that occurs in the canine population, yet little progress in changing outcome has been achieved in the past 20 years. While doxorubicin (DOX) based protocols have improved survival times in dogs with resectable tumors from an average of 3 months to 6 months, nearly all dogs die due to metastatic disease within 1 year of diagnosis, with most deaths occurring within 6–8 months [2]. A variety of chemotherapy combinations have been used, most of which are centered around the use of doxorubicin (DOX) including DOX/cyclophosphamide, DOX/cyclophosphamide/vincristine and liposomal DOX, however none of these have been found to be substantially better than the other [6, 8, 10, 11].

The current study was designed to evaluate the efficacy of toceranib against stage I and II splenic HSA in the microscopic disease setting following the administration of DOX chemotherapy. The underlying premise in support of our hypothesis was that inhibition of both PDGFR and VEGFR family members would have direct effects on tumor cell growth as well as indirect effects on the tumor microenvironment by virtue of presumed targeting of tumor vasculature. Therapies targeting VEGFR in people have largely resulted in modest benefits, and unfortunately, in this clinical trial, toceranib did not provide a measurable impact in dogs with splenic HSA with respect to either DFI or OS when compared to previous clinical investigations in this disease setting.

There have been several attempts to dissect the molecular mechanisms that drive HSA with the ultimate goal of improving therapeutic outcome. Multiple receptor tyrosine kinases are known to be expressed by HSA tumor cells including KIT, PDGFR, and VEGFR, although their contributions to tumor growth are not clear [17, 18, 20–23, 32]. The small molecule inhibitors imatinib, dasatinib, and masatinib have all shown activity against canine HSA cell lines in vitro, with evidence of direct effects on PDGFR family members [16, 19]. Furthermore, imatinib demonstrated activity against canine HSA mouse xenografts, supporting the role of PDGFR signaling on tumor biology in vitro [16]. A more recent immunohistochemical study found that only 45 % of tumors were positive for PDGFRα and 63 % positive for PDGFRβ; however there was no evidence of overexpression or activating receptor mutations in the tumor samples evaluated [33]. These data suggest that while PDGFRα/β expression may be detected in HSA, the receptors likely do not play a critical role in tumor growth, providing a possible explanation for why toceranib was not beneficial in the study population reported herein.

With respect to VEGF/VEGFR signaling, expression of both ligand and receptor have been demonstrated in tumor samples and cell lines providing some evidence that this signaling pathway may be relevant in HSA biology [18, 20, 22, 23]. While not yet demonstrated in the clinical setting, several murine xenograft studies have suggested that VEGFR inhibitors can promote a more aggressive phenotype through the process of metastatic conditioning [34–37]. This has raised concerns that the use of VEGFR inhibitors in the setting of microscopic disease could potentially accelerate the development metastasis. Mechanisms proposed to drive tumor progression and metastasis in the face of VEGFR inhibition include vascular pruning leading to acute hypoxic stress, activation of alternative angiogenic pathways (e.g. signaling between the tumor stroma and cells of the immune system) and up-regulation of proangiogenic factors (e.g. FGF, PDGF), among others [38–42]. Interestingly, one study found that VEGFR1 protein expression was higher in tumors derived from Golden retrievers, and that targeted inhibition of VEGFR1 increased proliferation of tumor cells derived from Golden Retrievers, but not from other breeds [21]. These findings highlight the notion that expression of a particular kinase does not necessarily define its role as a driver in tumor biology, and as such, inhibition of that kinase may have unexpected consequences, such as acceleration of tumor growth.

While VEGFR and PDGFR may contribute to tumor cell growth in HSA, it is possible that cytoplasmic signaling pathways downstream of these receptors are also dysregulated thus limiting the effect of upstream inhibition. For example, recently mitogen-activated protein/extracellular signal-regulated kinase (MEK) inhibitors reduced extracellular signal-regulated kinase (ERK) activation and the viability of primary cells derived from visceral, cutaneous, and cardiac HSA in vitro, indicating that this cytoplasmic kinase may be critical for sustained tumor cell growth [24]. These cells were also sensitive to sorafenib, an inhibitor of multiple receptor kinases including VEGFR, PDGFR, KIT as well as the cytoplasmic kinase B-RAF. In vivo, both MEK inhibitors and sorafenib decreased the growth of HSA xenografts in mice. Similar results were observed with human angiosarcoma cell lines and xenografts, supporting a role for the MEK signaling pathway in the biology of this disease in both species.

There are other possible explanations for the failure of toceranib to improve outcome in this clinical trial. Given the conflicting data regarding the roles of PDGFR and VEGFR family members based on immunohistochemical staining, cell line studies, genomic analysis and murine xenograft work, it is possible that these receptor tyrosine kinases simply do not drive HSA tumor growth or survival. As such, inhibiting these pathways would provide little benefit in dogs with disease. Alternatively, it may be possible that these pathways play a role early on in tumor cell growth and survival, but resistance to toceranib develops early on and as such, an effect on disease free interval is not observed. Lastly, there may be a limited window of opportunity to affect the growth of microscopic metastatic disease with toceranib and delaying treatment until after DOX rather than concurrent with DOX may have diminished any therapeutic effect. This may be particularly relevant for any anti-angiogenic effects as typical cytotoxic chemotherapy based regimens have little impact on angiogenesis mediated by circulating endothelial precursors thus potentially permitting microscopic disease to develop a more mature vasculature that would be resistant to toceranib treatment. Studies evaluating the impact of toceranib on circulating endothelial precursor levels during cytotoxic chemotherapy would be useful to determine if concurrent treatment might be of benefit in dogs with microscopic metastases.

In the present study, 7 dogs failed doxorubicin treatment either before completion of DOX (*n* = 3) or at re-staging (*n* = 4) and an additional 3 dogs were found to have metastatic disease immediately following initiation of toceranib therapy. With respect to dogs diagnosed with stage I HSA, 4 of 5 were removed from the study either prior to or soon after starting toceranib due to disease progression. Given the small number of dogs with stage I disease, statistically significant comparisons cannot be made, however dogs with stage I disease did not appear to have a survival benefit when compared to those with stage II HSA. The fact that 23 % of dogs developed disease recurrence within 3 months following surgery and treatment with DOX underscores the aggressive nature of this disease. Furthermore, the reported median DFI and ST reported herein do not represent an improvement upon previously published reports of adjuvant chemotherapy for splenic HSA [6, 8–10, 12]. It may therefore be of interest to determine whether the inclusion of toceranib during DOX treatment rather than following chemotherapy would provide a greater survival benefit.

The adverse events associated with toceranib administration were expected, consisting mainly of grade 1 and 2 gastrointestinal (GI) adverse events including inappetance, weight loss, vomiting and diarrhea. The grade 3 and 4 GI adverse events were also similar to those in previous studies [28–30]. In this study, 19 dogs had a dose reduction in toceranib secondary to the development of clinical toxicities and only 1 dog was removed from the study due to toceranib effects that could not be effectively managed with either a dose reduction or the use of concomitant medications. More recently, doses of toceranib between 2.5–2.75 mg were associated with biologic activity of drug, clinical benefit to treated dogs, and no grade 3 and 4 GI adverse events [43]. Therefore, any future studies evaluating the concurrent administration of toceranib in combination with doxorubicin or other chemotherapy agents could use this lower starting dose of toceranib and potentially reduce the risk for severe GI toxicities.

There are several weaknesses in this study that should be noted. Due to the multi-institutional nature of the study, there was likely inherent variability in data collection and interpretation and assessment of clinical toxicities. To help ensure uniformity in the data, patient AEs were assessed in accordance with VCOG-CTCAE criteria and the primary investigator (C. London) provided direction to all sites regarding management of AEs and recommended dosing of toceranib throughout the study period. In addition, this clinical trial lacked a prospective control group that received doxorubicin alone and was not randomized. Splenectomy and DOX-based chemotherapy is the standard treatment for splenic HSA in dogs, and the addition of chemotherapy results in median survival times of approximately 6–8 months [2]. Our results were compared to historical information published on dogs undergoing chemotherapy and splenectomy. Although there were many similarities between our patient population and the historical populations, it is difficult to make direct comparisons. Lastly, the presence of metastatic disease at staging was ambiguous in some cases, and may have resulted in enrollment of dogs with unconfirmed early metastatic disease. This was most evident in the stage I dogs, in which 4 of 5 dogs were removed from the study early on due to metastatic disease.

As previously discussed, it is possible that toceranib may provide a greater benefit when administered earlier in the treatment protocol. To address this, toceranib could potentially be given concurrently with the DOX. This may be advantageous as DOX has been shown to inhibit HIF1-α [44], thus offsetting the hypoxic effects of vascular pruning induced by VEGFR inhibition. MTD chemotherapy has been combined with toceranib in the treatment of some solid tumors [45, 46]. These studies showed that toceranib often sensitizes the myeloid compartment to the effects of cytotoxic chemotherapy resulting in severe neutropenia, necessitating dose reductions in the chemotherapy. If DOX were to be used concurrently with toceranib in dogs with HSA, it is probable that lower doses would be required to avoid severe adverse events, thus potentially mitigating the benefit of cytotoxic chemotherapy. Nevertheless, antitumor responses are still observed in this setting of combined chemotherapy/toceranib in which the chemotherapy dose has been reduced to below that typically used in treatment. For example, in dogs receiving combined vinblastine/Palladia for the treatment of mast cell tumors, the chemotherapy dose was decreased to 1.6 mg/m2 (standard dose is 2–2.5 mg/m2), yet the objective response rate in treated patients was 70 % [46].

## Conclusions

In summary, the data reported in this manuscript demonstrate that the use of toceranib following DOX chemotherapy does not improve either DFI or OS in dogs with stage I and II HSA. Given the early failure rate in a high percentage of dogs enrolled in this study, future studies with DOX and toceranib in addition to metronomic therapy with low-dose cyclophosphamide may be warranted to determine if concurrent rather than sequential administration of these therapies has a significant impact on disease progression in dogs with HSA.

## Abbreviations

HSA: Hemangiosarcoma
DOX: Doxorubicin
GI: Gastrointestinal
PDGFR: Platelet derived growth factor receptor
VEGFR: Vascular endothelial growth factor receptor
L-MTP-PE: Muramyl tripeptide phosphatidylethanolamine
FAK: Focal adhesion kinase
IACUC: Institutional animal use and care committee
CBC: Complete blood count
EOD: Every other day
AE: Adverse event
DFI: Disease free interval
OS: Overall survival
MEK: Mitogen-activated protein/extracellular signal-regulated kinase
ERK: Extracellular signal-regulated kinase
